# Correspondence, Intelligence, &c.

**Published:** 1822-09-01

**Authors:** 


### Correspondence, Intelligence, &c.

The epistle from Dr. L. Green, of Bethlem, Pensylvania, cost us 3*s.* 8*d.* postage. We are personally obliged to the Doctor for his kind expressions; but cannot approve of the specimens of his lucubrations which he has enclosed to us. They are very far from being creditable to any man who writes M. D. after his name. As for the attack which he laments as having been made on the Editor of this Journal, in the "*Great National Gazette*," Dr. Green is requested to make himself perfectly easy on that score, the Editor having great hopes that he will survive the shock, even without the assistance of Dr. Green's pen. *Non tali auxilio, &c.* A man's writings must defend themselves, and the laws will defend the writer.

The suggestions of a "constant reader" to republish the review he alludes to, in a separate form, cannot be complied with, especially as the article reviewed is announced for publication as a distinct work by the original author, who will probably avail himself of the observations we made on the subject.

"Crito" *may* be right in his strictures on * * * *, but he *may* be wrong, and in that case we should be *doubly so,* for giving them a currency, the effects of which might never be completely remedied. We cannot avail ourselves of any critical remarks that have not obviously the *public good* for their object and end.

*Medical Intelligencer.* In one of the late numbers of that work, the Editor states that we have *taken the plan of our Periscope directly from the Medical Intelligencer.* The *Periscope* was planned and executed precisely as it is at present, (as all our readers and all his readers very well know) so early as July 1818, a year, or a year and a half before the Medical Intelligencer was in existence. If the two plans, then, are so exactly similar, we leave the public and the editor himself to judge which of the two is likely to be the copy and which the original.

But if the editor had given himself a moment's time for consideration in comparing the two plans, he would never have said that one was a copy of the other. We shall point out three principal distinctions between them. In the first place, the Medical Intelligencer engages to give some account of *all* papers in *all* the periodicals connected with medicine.—The Periscope only notices such papers as suit its purpose. 2dly. A considerable portion of the Medical Intelligencer is occupied in *reviewing reviews.*—The Periscope never meddles with such subjects. 3dly. The *extent* of the Medical Intelligencer's plan necessarily confines it to little more than a sketch of each article.—The plan of *selection* on which the Periscope is conducted, enables it to give the sum and substance of almost every article to which it directs its attention. Surely these are distinctions which destroy every thing like identity, or even analogy of plan or execution in the two works.

The letter of Mr. G———, from Brighton, came to hand; but we are unwilling to harrow up the feelings of the public by the exposure of such ignorance and negligence as Mr. G. points out. In so extended a profession as ours, and where there are so few legislative regulations, there must always be a considerable number of ignorant and unprincipled practitioners—and that even within the pale of regular medical society. But we do believe that the proportion of these is daily lessening, and that the diffusion of knowledge is daily exposing and beating back these pretenders to science.

*Apothecaries' Act.* We are glad to observe that this act now begins to wear an operative aspect. A *soi disant* Dr. Bastow, residing at Halifax, in Yorkshire, has been prosecuted by the Company, and fined in a penalty of 20*l.* which will most likely check his career in the Doctorate. This worthy disciple of Esculapius was originally a stable-boy to a surgeon, in which situation he probably doctored the horses' heels, or might even carry out the medicines occasionally. He then turned to the trade of *card-making*, but was promoted, in the year 1820, to the rank of a common foot soldier, in which he continued till he commenced *business* as a doctor-apothecary in Halifax. Here he began to cure consumption in males by his "morning honey," and in females, by his "evening honey," with a wee drap of gin in it, (which he charged, by the way, as *brandy*, though the difference of price is well known) acquiring great reputation, notwithstanding his patients were "Yorkshire too," as a very skilful doctor! It is quite evident, however, that this hopeful youth must play his *cards* in another manner for the future.

*Dr. Pemberton.* The sufferings of this distinguished physician have terminated with his existence. It is well known to the profession that, for many years, he was a martyr to that painful disease, tic douloureux, for which every remedy had been tried in vain, including the division of the nerves by the knife. He lately paid the debt of Nature, worn out, we believe, by the harrassing attacks of this terrible malady. The principal, if not the only, morbid appearances on dissection, were, we are informed, a small spicula of bone projecting over the longitudinal sinus, a calculus in the gall-bladder, and an ounce of water in the lateral ventricles of the brain.

*Dr. Parry's Elements of Pathology.* Such has been the demand, we hear, for this work, since our analysis of it, that it is now completely out of print. We hope this will stimulate the younger Parry not only to reprint the first volume, but edit the remainder from his illustrious father's notes.

*Hamilton's Principles of Medicine.* The long, able, and severe review of this work came safely to hand from G———. We are sorry we cannot avail ourselves of the labours of our friends the reviewers (for there was a board) on this occasion, because, although we agree with them that "judex damnatur cum nocens absolvitur," yet we cannot, consistently with that duty which we owe the public, dedicate 20 pages to the condemnation of a work, the only evil attending which, is the loss of the *purchase money*—for no man will ever waste *time* in perusing it.

*The Nursery Guide.* We have received Mr. F's witty review of this curious production, but dare not venture on its insertion, notwithstanding the poetical taste of the times. The subject matter of this review was not worth the talent and labour expended in the critique—*materiam superabat opus.*

A Physician in the West End of the Town is desirous of receiving into his family two House Pupils, and from the nature of his own pursuits, embracing equally the literature and practice of his profession, he presumes that this opportunity may be worth the attention of industrious and liberally educated medical students.

Reference for particulars may be made to Dr. J. Johnson, Spring Gardens, who will give the address of the physician abovementioned.

*N. B. To prevent misapprehension, it may be proper to state that Dr. Johnson does not take pupils himself.*

---

*London Hospital Medical School.*

The Winter Course of *Lectures* will commence on Tuesday, the 1st of October, at Two o'Clock.

Theory and Practice of Medicine, by Dr. Robinson.

Materia Medica, and Institutes of Medicine, by Dr. Billing.

Midwifery and Diseases of Women and Children, by Dr. Ramsbotham.

General and Pharmaceutic Chemistry, by Dr. Gordon.

Clinical Lectures, by Dr. Robinson and Dr. Billing.

Anatomy, Physiology, &c. by Mr. Headington.

Principles and Practice of Surgery, by Mr. Headington.

Practical Anatomy and Demonstrations, by Mr. Luke.

*Further particulars may be obtained by application to Mr. Ward, Apothecary at the Hospital.*

---

*Medical School of Guy's Hospital.*

The Autumnal Course of Lectures will commence the 2d Oct. viz.

*Practice of Medicine*, by Dr. Cholmeley.—*Chemistry*, by Mr. Allan, Dr. Bostock, and Mr. Aikin.—*Experimental Philosophy*, by Mr. Allen and Professor Millington.—*Theory of Medicine, and Materia Medica*, by Dr. Cholmeley and Dr. Back.—*Midwifery and Diseases of Women and Children*, by Dr. Haighton and Dr. Blundell.—*Physiology, or Laws of the Animal Œconomy*, by Dr. Haighton, and Dr. Blundell.—*Structure and Diseases of the Teeth*, by Mr. Bell.—And a Course of *Practical Botany* in the Spring by Mr. Salisbury, late of the Botanic Garden, Chelsea.

N. B These several Lectures, with those *on Anatomy*, and on the *Principles and Practice of Surgery*, given at the Theatre of *St. Thomas's Hospital* adjoining, are so arranged, as not to interfere with each other in the hours of Attendance nor with the Medical or Surgical Practice of the Hospital: and the whole is calculated to form *A Complete Course of Medical and Chirurgical Instruction.* Terms and other Particulars may be learnt from Mr Stocker, Apothecary to *Guy's Hospital; who alone* is empowered to receive entrance money from Pupils, for any of these Lectures delivered at *Guy's Hospital.*

---

MEDICAL PUPIL.

A Physician attached to several Public Institutions is desirous of accommodating a respectable House Pupil. The terms and peculiar advantages of the residence here proposed may be known by reference to Dr. James Johnson, who will give the necessary address.

